# Silencing Glypican-1 enhances the antitumor effects of Pictilisib via downregulating PI3K/Akt/ERK signaling in chemo-resistant esophageal adenocarcinoma

**DOI:** 10.1080/23723556.2023.2238873

**Published:** 2023-08-15

**Authors:** Akshay Pratap, Andrea Qualman, Hedlund Garrett, Lindsey Westbrook, Erlinda The, Sanchayita Mitra, Mila Cordero, Kenneth Meza Monge, Juan- Pablo Idrovo, Argudit Chauhan, Linling Cheng, Mitchell Jay Cohen, Benedetto Mungo, Sachin Wani, Robert Alexander Meguid, Martin D McCarter, Xianzhong Meng

**Affiliations:** a Division of Gastrointestinal Tumor and Endocrine Surgery, University of Colorado, Aurora, CO, USA; b Department of Allergy and Clinical Immunology Flow Core, University of Colorado, Aurora, CO, USA; c Department of Pathology, University of Colorado, Aurora, CO, USA; d Division of Cardiothoracic Surgery, University of Colorado, Aurora, CO, USA; e School of Science, Engineering, & Technology, St. Mary’s University, San Antonio, TX, USA; f Department of Biomedical Engineering, University of Colorado, Boulder, USA; g Division of Surgical Oncology, University of Colorado, Aurora, CO, USA; h Division of Gastroenterology and Hepatology, University of Colorado, Aurora, CO, USA

**Keywords:** Glypican-1, Poorly differentiated esophageal adenocarcinoma (PDEAC), PI3K/Akt pathway, pictilisib, chemoresistance

## Abstract

Poorly differentiated esophageal adenocarcinoma (PDEAC) has a dismal prognosis. Glypican-1(GPC-1) is known to be upregulated in several cancer types in contrast to healthy tissues, rendering it as a biomarker. Nevertheless, the potential therapeutic targeting of GPC-1 has not been explored in PDEAC. There is accumulating evidence that GPC-1, via upregulation of PI3K/Akt/ERK signaling, plays a crucial role in the progression and chemoresistance in cancer. Pictilisib, a class I pan PI3K inhibitor, has shown promising antitumor results in clinical trials, however, has not gained widespread success due to acquired drug resistance. This study investigated the role of GPC-1 in chemo-resistant PDEAC and appraises the impact of targeted silencing of GPC-1 on the antitumor effects of Pictilisib in PDEAC cell lines. Immunohistochemistry assays in PDEAC tissue specimens demonstrated a pronounced intensity of staining with GPC-1. Upregulation of GPC-1 was found to be correlated with advanced stage and poor prognosis. In-vitro studies examined the influence of GPC-1 knockdown and Pictilisib, both as individual agents and in combination, on cytotoxicity, cell cycle distribution, apoptosis, and gene expression profiles. Silencing GPC-1 alone showed significantly reduced cell viability, migration, colony formation, epithelial-mesenchymal transition, and stemness in PDEAC cells. Significantly, knockdown of GPC-1 combined with low-dose Pictilisib led to enhancement of cytotoxicity, cell cycle arrest, and apoptosis in ESO-26 and OE-33 cells. In the xenograft mouse model, the combination of Pictilisib and GPC-1 knockdown exhibited synergy. These findings suggest that GPC-1 represents a promising target to augment chemosensitivity in esophageal adenocarcinoma.

## GRAPHICAL ABSTRACT

**Figure:**
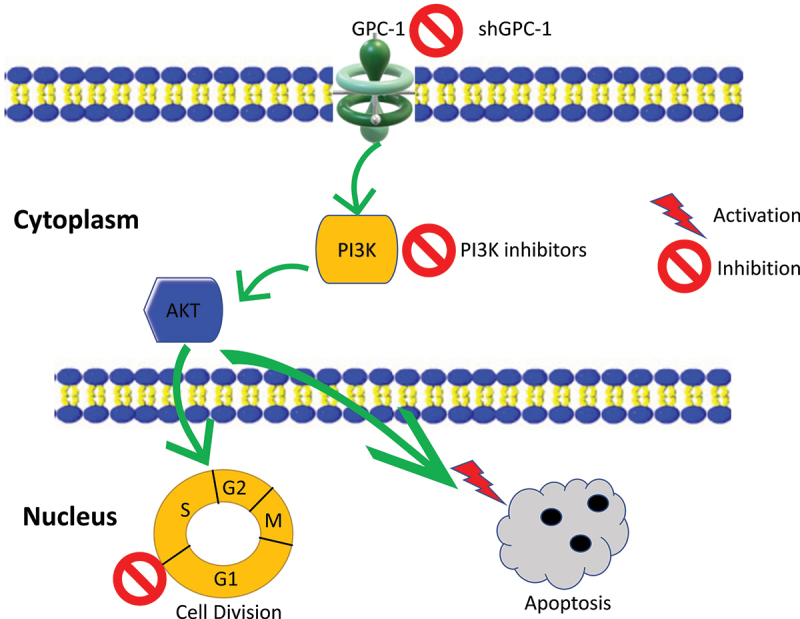

## Introduction

Esophageal cancer (EC) is one of the most malignant cancers which accounted for 0.54 million deaths worldwide in 2020.^1^ Esophageal adenocarcinoma (EAC), a subtype of EC commonly seen in the Western world, is growing exponentially due to rising prevalence of obesity and gastroesophageal reflux disease. It is often diagnosed in an advanced stage, shows rapid disease progression, frequent resistance to conventional chemotherapy and radiation which results in an extremely poor prognosis.^2,3^ Despite significant treatment advances the 5-year survival of EAC patients is only 23%.^4^ As our understanding of the molecular mechanisms underlying the biology of EAC improves, opportunities to develop novel effective therapies targeted toward improving survival and quality of life in EAC need to be explored. In recent year’s identification of molecular targets controlling tumor cell survival, proliferation and metastatic behavior have brought about a paradigm shift in precise medicine. However the heterogeneity in the landscape of EAC still poses a formidable challenge to design effective targeted therapy.^5^ Genetic mutations involving PI3K/Akt signaling are regarded as one of the most common drivers in cancers including EAC.^6,7^ Interestingly, analysis of TCGA database reveals amplification of PI3K/Akt, in 30% of locally advanced PDAEC.^8^ The PI3K/Akt crucially regulates several intracellular pathways to sustain cell growth, motility, survival, invasion and angiogenesis thereby making this pathway a potential target for therapeutic intervention.^9^ Not surprising several PI3K inhibitors have entered various stages of clinical trials often combined with chemotherapy.^10–12^ Pictilisib (GDC-0049) a pan PI3K inhibitor is of clinical relevance in the context of PI3K mediated development of cancer. Pictilisib was shown to have acceptable safety profile and tolerability in human trials however had modest clinical efficacy, a theme shared by all PI3K inhibitors.^13,14^ Two important lessons were learnt from clinical trials. Firstly, despite vertical blockade of PI3K/Akt axis with PI3K inhibitors activation of feedback loops stimulating Akt and its downstream m-TORC activation leads to uncontrolled tumor growth and resistance to PI3K treatment. Secondly, monotherapy with PI3K inhibitors inevitably has limited efficacy underscoring the importance of combination treatment with either chemotherapy or immunotherapy to yield their full therapeutic potential.^15,16^ These challenges associated with PI3K inhibition have led to an era of combination therapies to offer more effective tumor control. Furthermore, since cancers involves aberrant regulation of various signal transduction pathways, simultaneous inhibition of multiple deregulated targets is a promising therapeutic strategy.^17^ In a recent study our group identified critical role of Glypican-1 (GPC-1) in progression of EAC.^18^ GPC-1 is a member of the family of glycoproteins known as membrane heparan sulfate proteoglycans (HSPG).^19^ Glypicans serve as co-receptors to activate multiple intracellular signaling pathways which drive tumorigenesis, chemoresistance, angiogenesis, and metastasis.^20,21^ GPC-1 overexpression has also been linked to poor prognosis in esophageal cancer, pancreatic adenocarcinoma, and glioblastoma by promoting chemoresistance.^22–24^ Preclinical data has shown excellent antitumor efficacy of antiGPC-1 antibody drug conjugate in stroma rich pancreatic adenocarcinoma and cholangiocarcinom.^25,26^ Recently, Miltuximab, a chimeric antiGPC-1 antibody demonstrated excellent clinical efficacy in the first human trial in prostate cancer and has opened avenues for its use in other GPC-1 over expressing solid tumors. Based on these encouraging reports we hypothesize that targeting GPC-1 alone or in combination with PI3K inhibitors may offer an attractive alternative strategy to improve drug sensitivity and overcome drug resistance in refractory cancers such as EAC. In the present study, we investigated the significance of PI3K/Akt/ERK in poorly differentiated chemo-resistant esophageal adenocarcinoma (PDEAC) and explored the molecular mechanism of antitumor effects of GPC-1 knockdown alone or in combination with Pictilisib using *in vitro* and preclinical *in vivo* tumorigenesis model.

## Material and methods

### Chemical reagents

All chemicals and reagents were purchased from Millipore Sigma (St Louis, MO). GDC-0971 (Pictilisib) was purchased from Selleck Chem (Houston, TX, USA). Dimethyl sulfoxide was used to reconstitute GDC-0971.

## Human tissue samples

In this study, a sample size comprising 25 pairs of poorly differentiated esophageal adenocarcinoma tissues (PDEAC) and their respective adjacent non-tumor tissues were collected. These specimens were obtained from a cohort of 25 patients who underwent surgical resection of the tumor at the University of Colorado during the period spanning from 2019 to 2022. The patient’s final pathology was interpreted as minimal or no response to treatment. All patients had received neoadjuvant chemotherapy according to NCCN guidelines for esophageal adenocarcinoma.^27^ The research under consideration received the necessary approvals from the Ethics Committee of University of Colorado (IRB#19–1319). The experimental work with human tissue samples was conducted in accordance with the Declaration of Helsinki (as revised in 2013). Prior to enrollment in the study, all participants provided informed consent.

## Cell lines culture and transfection

Cell lines were purchased from American Type Culture Collection (Manassas, VA, USA). Normal human esophageal epithelial HET1A cells were grown in complete Bronchial Epithelial Growth Media (Lonza, Basel, Switzerland). EAC cell lines (ESO-21, ESO-56, OE33, OE19, and SKG-T4) were grown in Roswell Park Memorial Institute Medium (RPMI 1640; Gibco, Grand Island, NY), FLO1 and HEK 293T cells were grown in Dulbecco’s Modified Eagle Medium (DMEM, Gibco, Grand Island NY). Growth media was supplemented with 10% fetal bovine serum (FBS), 0.5% penicillin/streptomycin, and gentamicin/amphotericin (1:500 dilution). All cells were incubated in a humidified atmosphere with 95% air and 5% carbon dioxide at 37°C. Serum-reduced media for lentivirus transfection consisted of growth medium with 2–5% FBS, 0.5% penicillin/streptomycin, and gentamicin/amphotericin (1:500 dilution).

## Transfection and establishment of stable cell lines

Primers and restriction enzymes were purchased from Integrated DNA Technology and NEB Bio labs. For GPC-1 knockdown, three unique shRNA clones (shGPC-1α, shGPC-1-β, and shGPC-1γ) were designed based on the human GPC-1 gene (GenBank accession number: NM https://www.ncbi.nlm.nih.gov/gene/2817). A scrambled shRNA was designed and synthesized for negative control (SCR). The primer sequences for the shRNA clones are shown in Table 1. The primer oligonucleotides were annealed using the following parameters: 80°C for 2 min, 65°C for 10 min, 37°C for 10 min and 25°C for 5 min. The PCR product was gel extracted, purified, and cloned into the psi-LVRH1GH vector (Genecopeia, MA, USA) using easy cloning kit (New England Bio labs, MA, USA). GPC-1 overexpression plasmid was constructed by cloning the coding sequence of GPC-1 gene into pEZ-Lv201(Genecopeia, MA, USA) at Bam HI/Eco RI site on the vector using easy cloning kit (New England Bio labs, MA, USA). The vectors carried an extended green fluorescent protein (e-GFP) reporter gene driven by SV-40. The ligated products were transformed into competent DH5α cells (Zymo Research, Irvine, CA, USA) using a heat shock method. The transformed cells were grown on an LB-agar plates containing ampicillin at 37°C and 5% CO_2_ overnight. The overnight culture was used for plasmid extraction using commercially available ZymoPURE Plasmid Miniprep kit (Zymo Research, Irvine, CA) per manufacturer’s instructions. GPC-1 knockdown and GPC-1 overexpressed clones were verified by restriction digest analysis on a 1.3% agarose gel. Lentivirus were prepared using lentivirus packaging plasmid kit (Genecopeia, WA, MA) using the manufacture’s protocol. HEK 293T cells were seeded in 6 well plates and transfected with packaging plasmid and constructs. Lentivirus was collected 48 h later. Stable cell lines with GPC-1 knockdown and overexpression were selected with puromycin 1ug/ml for ESO-26 and OE-33 cells and 0.5ug/ml for SK-GT4 cells.

**Table 1.** Sequence of GPC-1 shRNA and GPC-1 overexpression plasmid.

Clone	Gene	Location	Length	Sequence
shGPC-1α	GPC-1 (NM_002081.2)	2,965	21	CCAAACATGCATCCATTTACT
shGPC-1β	GPC-1 (NM_002081.2)	956	21	GTGCTCGAGAGCTGTCATGAA
shGPC-1Δ	GPC-1 (NM_002081.2)	1,029	21	GACTATTGCCGAAATGTGCTC
SCR			19	GCTTCGCGCCGTAGTCTTA
EV	GPC-1 (NM_002081.2)	1,677	24	GATAGCACTGAGCACCTGTTCCAG

## Quantitative real-time PCR

Quantitative real-time PCR (qRT-PCR) was performed in 96-well plates using the iQ SYBR Green Supermix (Bio-Rad, Hercules, CA) and Roche Light Cycler 96. Total RNA was extracted from cells using the Trizol reagent (Thermo Fisher Scientific, Waltham, MA). Next, 1ug of RNA was reverse-transcribed into cDNA using the All-in-One First-Strand cDNA Synthesis Supermix kit (Applied Biological Materials, Richmond, BC, Canada). Relative expression of target genes was calculated with the 2^−ΔΔCt^ method based on Ct values, using β-actin as internal control. Primer sequences are listed in Table 2.

**Table 2.** Primer sequences.

Gene	Sequence
GPC-1	Forward sequence: 5’-TGAAGCTGGTCTACTGTGCTC-3’
	Reverse sequence: 5’-CCCAGAACTTGTCGGTGATGA-3’
Beta actin	Forward sequence: 5’-TTGGCCAGGGGTGCTAAG-3’
	Reverse sequence: 5’-AGCCAAAAGGGTCATCATCTC-3’

## Western blotting

Cells were washed with ice-cold phosphate-buffered saline (PBS) and then lysed with ice-cold RIPA buffer (Sigma Aldrich, St Louis, MO, USA). Protein concentration was determined using BCA assay (DC assay, Bio-Rad, Hercules, CA) using bovine serum albumin to generate a standard curve. Equal amounts of the protein concentrations (25ug) were separated using sodium dodecyl sulfate-polyacrylamide gel (Bio-Rad 4%–20%) electrophoresis and transferred to 0.4um nitrocellulose membranes. Membranes were blocked in TBS-Tween 20 with 5% nonfat milk for 1 h. Antibodies and dilutions are listed in Table 3. Primary antibodies were diluted in 5% BSA and incubated at 4°C overnight. Secondary antibodies were diluted in TBS-Tween 20 with 5% nonfat milk. Protein quantification with densitometry analysis was performed using Image Lab Software (Bio-Rad Laboratories, Inc. 2017).

**Table 3.** List of antibodies.

Antibody	Manufacturer	Catalogue	Concentration
Glypican 1	Abcam	ab199343	1:750
ZEB-1	Proteintec	21544–1-AP	1:1000
N-Cadherin	Cell Signaling	13116S	1:1000
E-Cadherin	Cell Signaling	3195	1:1000
c-myc	Cell Signaling	18583	1:1000
KLF-4	Cell Signaling	12173	1:1000
Nanog	Cell Signaling	8822	1:1000
OCT-4	Cell Signaling	2750	1:1000
p-PI3Kp85	Cell Signaling	17366	1:1000
PI3K	Cell Signaling	4292	1:1000
pAkt(Ser 473)	Cell Signaling	4060	1:1000
p-PRAS40(Thr 476)	Proteintec	29072–1-AP	1:1000
PRAS40	Proteintec	21097–1-AP	1:1000
p-P70S6K (Thr 379)	Proteintec	28735–1-AP	1:1000
P70S6K	Proteintec	66638–1-Ig	1:1000
p-ERK1/2	Cell Signaling	Cell Signaling	1:1000
ERK1/2	Cell Signaling	Cell Signaling	1:1000
AKT	Cell Signaling	4691	1:1000
F-Actin	Abcam	ab130935	1:1000
SNAIL 1	Proteintec	13099–1-AP	1:1000
Vimentin	Proteintec	10366-AP	1:1000
Beta Actin	Proteintec	66009–1-Ig	1:10,000
Secondary HRP Rabbit	Proteintec	SA-00001-2	1:5000
Secondary HRP Mouse	Proteintec	SA-00001-1	1:3000

## Cell proliferation CCK-8 assay

Cells were seeded in 96 well plate in triplicate. Plates were treated with CCK-8 solution (GlpBio, Montclair, CA) at various time points. Plates were incubated for 2 h and read on BioTek microplate reader. The OD values (450 nm) were measured, and growth curve constructed.

## Flow Cytometry for cell cycle phases and apoptosis

Cells were seeded in 24 plates at a density of 200,000 cells per well for 24 h in antibiotic-free media. Cells were harvested for cell cycle status using a Propidium Iodide Flow Cytometry Kit (ab139418; Abcam, Cambridge, UK). Samples were analyzed by flow cytometry using a BD FACS Calibur flow cytometer (BD Biosciences, San Diego, CA). For apoptosis, cells were seeded into 24 well plates at 200,000 cells per well and allowed to adhere for 24 h. Cells were prepared for flow cytometry using Tonbo Bioscience PE Annexin V Apoptosis Kit (Tonbo Bioscience, San Diego, CA). Samples were analyzed using a BD FACS Canto II. Data were analyzed using Flowjo v.10.8 software.

## Transwell migration and invasion assay

Cells were seeded at a 5 × 10^5^ cells per well in polycarbonate filters with 8-μm pores (Corning Costar) combined with 24‐well culture plates were used for migration (uncoated) and invasion (Matrigel‐coated) assays. Cells (8 × 10^5^ cells/mL) were collected and resuspended in 100 µL serum‐free DMEM. Then, the cells were seeded on each polycarbonate filter in the 24‐well plates, and the bottom chambers contained 600 µL 20% FBS‐DMEM. After the incubation at 37°C for 24 h, the cells were fixed in 4% paraformaldehyde and stained for 30 min in a 0.1% crystal violet solution in PBS. The number of cells on the underside of each insert was determined using light microscopy (Nikon Ti2 microscope). Five randomly selected fields were counted per insert.

## Colony formation assay

Stably expressing shSCR and shGPC-1β ESO-26 and OE-33 cells were seeded into 6 well plates (density of 5000 cells). Cells were treated with Pictilisib at concentrations of (0, 0.1,1,5 and 10 µM) for 48 h. Medium was replaced with fresh 10% medium and cells were cultured for additional 14 days. Cells were fixed with ice-cold 4% paraformaldehyde and stained with 0.1% crystal violet. The colony numbers were then counted in random 10 high power fields using NIS image software on Nikon Ti2 microscope.

## Immunohistochemistry

5 µm tissue sections were prepared from paraffin-embedded blocks. Tissue sections were deparaffinized with xylene and rehydrated with graded ethanol. Anti-GPC-1 antibody staining was performed using Rapid IHC kit (Bio Vision) according to the manufacturer’s protocol. Immunostaining was scored as: 0, no staining; 1, normal staining; 2, strong staining. The ‘density’ of staining (termed the positivity score) was as follows: 1, indicates less than 50% positivity; 2, indicates more than 50% positivity. The final IHC score was determined by multiplying the intensity score by the positivity score, with a maximum positive score of 4. These data were called the GPC-1 H score to categorize them into low and high expression groups.

## Immunofluorescence and proximity ligation assay

For immunofluorescence staining, approximately 1 × 10^5^ cells were seeded on cover slips. After 24 h, cells were washed with PBS and fixed with 4% PFA followed by permeabilization with 0.1% TritonX. Cells were blocked in 0.1% BSA for 1 h and then incubated with primary antibodies at 4°C overnight. Secondary antibodies were applied prior to visualization under a fluorescent microscope (Nikon, TE2000). Proximal ligation assay was performed using a commercially available kit (Duolink, Sigma Aldrich, MO) following the manufacturer’s protocol. After incubation with PLA blocking solution, EAC cells were incubated with primary anti – GPC-1 (1:100 dilution) and anti- β tubulin antibody (1:100 dilution). The cells were incubated with the PLA probes, anti-rabbit PLUS, anti-mouse MINUS, washed, ligated, and amplified by rolling circle amplification. Images were obtained with a fluorescent microscope (Nikon, TE2000).

## TUNEL analysis

Apoptotic DNA fragmentation was detected using double fluorescence with CF-594 labeling terminal UTP-nick end labeling kit (Biotium, Fremont, California, USA) and 4’,6-diamidino-2-phenylindole (DAPI; Thermofisher, Waltham, MA, USA). Cells were imaged under a fluorescent microscope (Nikon, TE2000).

## Xenograft model

Stable shGPC-1 or Scramble transfected ESO-21 cells (1 × 10^6^ cells) were injected into the flank of BALB/c male nude mice (four per group) subcutaneously. Xenografts were assessed for their growth biweekly. Tumor volume was calculated using the formula; tumor volume (mm^3^ =/6 × a × b.^2^) When tumor volumes reached 50 mm^3^ mice were randomly divided into four groups (4 mice per group). Group 1: scramble control; Group 2: shGPC-1, Group 3: Scramble control +Pictilisib; Group 4: shGPC-1+ Pictilisib. Pictilisib was reconstituted in 30% PEG-300 with 1% Tween80 and 1%DMSO. Mice of treatment group were dosed with 100 mg/kg Pictilisib by oral gavage daily for the duration of the study while mice of untreated group received same amount of PBS saline by oral gavage. The animals were observed for 15 days after tumor cell implantation. After 15 days, tumor xenografts were harvested and analyzed with immunohistochemical staining for Ki-67 and TUNEL assay as well as western blot for protein analysis. All the in vivo experiments were approved by the Institutional Care animals at University of Colorado (IUCAC number: 1202)

## Public data sets

Publicly available data set was accessed to retrieve expression profiles, clinicopathological data, and survival of patients with EAC and non-tumor tissues (The Cancer Genome Atlas TCGA) repository, which is accessible at https://portal.gdc.cancer.gov/).^28^ The parameters for the incorporation of clinical information in the study were established as follows: (1) patients were deemed eligible if their clinical data were comprehensive; and (2) specimens with a follow-up duration of over 30 days were admitted. GSEA analyses for Gene Ontology (GO) enrichment analysis was performed using Correlation AnalyzerR.^29^ The *P* value was corrected by the Benjamin – Hochberg method, with a *P* value < 0.05 and a q value < 0.05 being the cutoff criteria. Survival analysis was downloaded from Kaplan Meier plotter and TCGA database (https://kmplot.com/analysis/).

## Statistical analysis

All experiments were performed at least three times. Clinicopathological variables were compared using the chi-square test. Data are presented as mean ±standard deviation and comparisons made using student’s t-test. Data with multiple comparisons were analyzed by ANOVA followed by Fisher’s least significant difference post hoc test. Chi-square tests or Fisher’s exact tests were used to assess the correlation of GPC-1 with clinical pathological characteristics and Student’s t test was used for comparisons between two groups.  All statistical analysis was performed using Graph Pad prism software. A *p* value < 0.05 was considered statistically significant.

## Results

### Expression of GPC-1 is upregulated in esophageal adenocarcinoma

**Figure 1.**
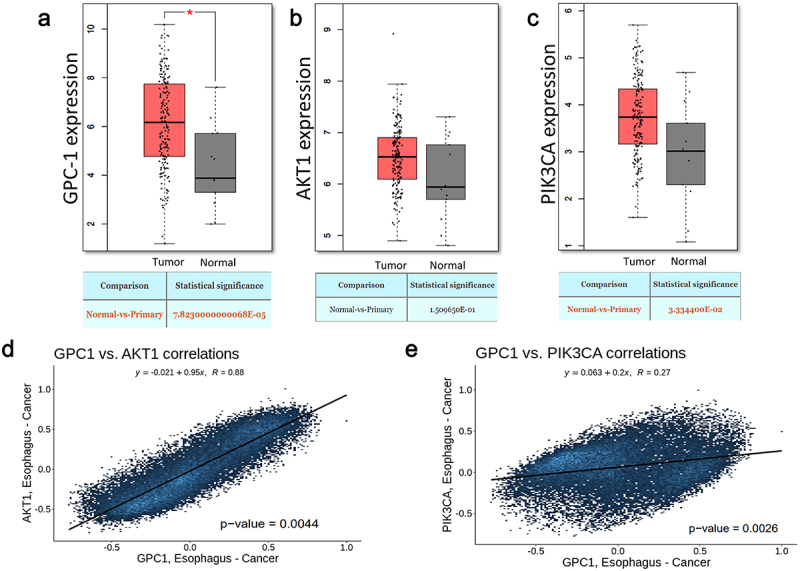
Expression of GPC-1 and PI3K/Akt is upregulated in esophageal adenocarcinoma.(a-c) GPC-1, Akt, and PI3K expression in tumor (*n* = 230) and paratumor tissue (*n* = 135) samples in TCGA project through the utilization of Student’s t-test. The tumor tissues are marked with red color and paratumor tissues are denoted by gray color. (d-e) Pearson’s correlation of GPC-1 expression with Akt and PI3K in TCGA project.

To elucidate the role of GPC-1 and PI3K/Akt signaling in EAC, we initiated the study by evaluating the mRNA expression levels of GPC-1, PI3K/Akt in EAC tumor tissues through publicly accessible datasets. The analysis unveiled a substantial upregulation of GPC-1 and PI3K/Akt in EAC tumors in comparison to adjacent normal tissues available in the Cancer Genome Atlas (TCGA) and Genotype-Tissue Expression (GTX) datasets (Figure 1a-c). The expression of GPC-1 had a positive correlation with Akt and PI3K expression (Figure 1d, e).

### Expression of GPC-1 is upregulated in chemo resistant PDEAC

**Figure 2.**
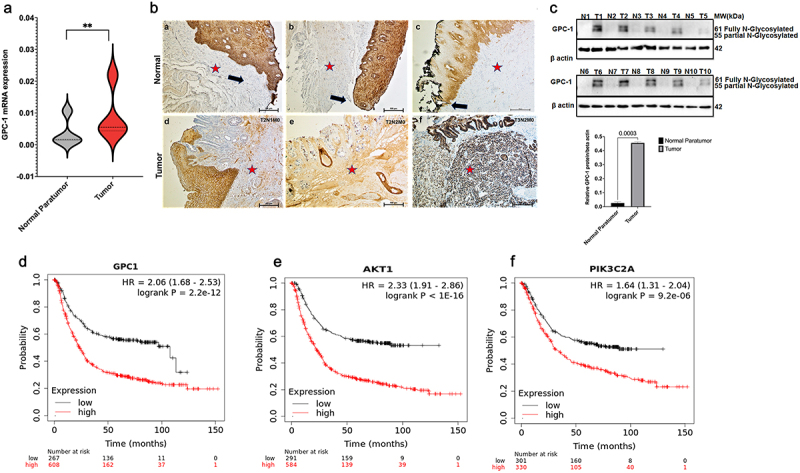
Expression of GPC-1 is upregulated in poorly differentiated esophageal adenocarcinoma. (a) Esophageal adenocarcinoma (EAC) tissues were observed to manifest an upregulation of GPC-1 expression compared to normal para tumor tissue in 25 paired tissues with poorly differentiated chemoresistant EAC at the University of Colorado. (b) Representative IHC photographs of normal paratumor gastroesophageal junction stained for GPC-1 (a-c) and tumor tissue stained for GPC-1(d-f) showing GPC-1 staining of normal paratumor gastroesophageal junction (black arrows). GPC-1 immunoreactivity is seen in basal and suprabasal layers of normal stratified epithelium. The normal paratumor stroma (red star), crypt epithelium, or columnar epithelium does not stain with GPC-1. Moderate to intense staining is noted in the stroma and columnar epithelium (red star). (c-d) Real-time q-PCR and western blot analysis of GPC-1 mRNA and protein in 10 paired tumor and paratumor tissue. The bar chart represents mean ±SD, *n* = 3. **, P < 0.01.

To verify the above assertion, an examination of GPC-1 mRNA expression was conducted on a sample collection consisting of 25 pairs of tumor tissue and adjacent paratumor normal tissue from poorly differentiated EAC patients who had undergone surgery and their final pathology revealed minimal to no treatment effect. The results produced consistent findings of increased GPC-1 mRNA in tumor tissues compared to normal paratumor tissue (Figure 2A). We next undertook an evaluation of GPC-1 protein expression in 25 PDEAC paraffin embedded tissues using immunohistochemistry. In the normal paratumor gastroesophageal junction, GPC-1 staining was seen in basal and suprabasal layers of stratified squamous epithelium (Figure 2B). GPC-1 is localized to the cytoplasm and cell membrane. Of note, there is no immunoreactivity of GPC-1 in the stroma or columnar. On the contrary, expression of GPC-1 in tumor tissue was significantly higher in the stroma and glandular epithelium (16/20, 80%) compared to normal paratumor tissue (2/20%). Furthermore, the western blot was performed in a representative set of 10 paired PDEAC and adjacent normal tissue. Results showed significantly higher expression of GPC-1 protein in PDEAC tissue compared to normal paratumor tissue (Figure 2C). We further correlated the expression of GPC-1 with patient’ pathologic characteristics (Table 1). The results indicated that the expression of GPC-1 was positively correlated with TNM stage (*p* < .05) and lymph node metastasis, Table 4. Small sample size in our study precluded analysis of survival, however analysis of prognostic data conducted utilizing TPM and FPKM classifications from the TCGA database yielded results indicating that overexpression of GPC-1 and PI3K/Akt was significantly associated with an unfavorable prognosis among patients with EAC (Figure 2d-f). The overexpression of GPC-1 and PI3K/Akt exhibited a significant correlation with a reduced overall survival rate among patients diagnosed with EAC. This statistical observation indicates the detrimental impact of GPC-1 and PI3K/Akt overexpression on the clinical outcomes of EAC patients.

**Table 4.** Univariate and multivariate analysis of prognostic variables in chemo-resistant PDEAC.

Variable	Univariate Analysis			Multivariate Analysis		
	P value	HR	95% CI	P value	HR	95% CI
GPC-1 expression (Low/High)	0.001**	1.22	1.116–2.121	0.001**	1.02	1.001–2.130
TNM stage(I+II/III+IV)	<0.001	2.233	1.744–2.912	0.002	1.31	1.012–2.301
T stage(T1+T2/T3+T4)	<0.001**	2.129	1.243–3.554	0.04*	1.68	1.002–2.650
N stage(N0/N1+N2+N3)	<0.001**	2.432	1.732–3.102	0.001**	1.878	1.221–2.602
Age (<60/>60)	0.34	1.045	0.786–1.478			
Sex (Male/Female)	0.26	1.066	0.810–1.430			

### Expression and localization of GPC-1 in PDEAC cell lines

**Figure 3.**
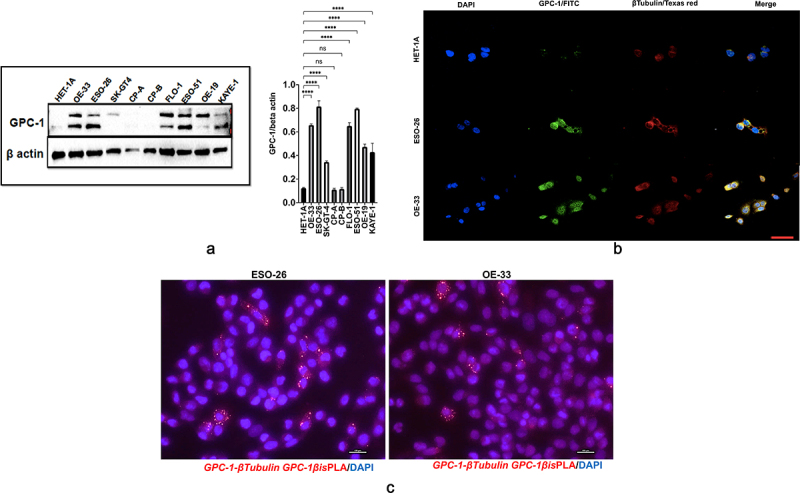
Expression and localization of GPC-1 in PDEAC cell lines. (a) Western blot analysis of GPC-1 expression in seven PDEAC cell lines compared to normal esophageal cell line HET-1A and Barrett’s cell lines (CP-A and CP-B). Bar chart of densitometry analysis of GPC-1 protein expression in PDEAC cell lines. (b) Representative immunofluorescence staining of normal HET-1A cells and PDEAC cells (ESO-26, OE-33). Cells were stained with DAPI (blue), GPC-1(FITC), and Tubulin (Texas red). The scale bar represents 50 µm. (c) Representative images of *insitu* Proximity ligation assays (PLA) of two EAC cell lines with anti GPC-1 and anti β-tubulin antibodies. Scale bar 100 µm. DAPI, 4’,6-diamidino-2-phenylindole; FITC, fluorescein isothiocyanate; ns; not significant, **** *p* < .0001.

We next analyzed GPC-1 expression in 7 EAC cell lines (OE19, OE33, FLO1, ESO21, ESO56, and SK-GT-4) which have been derived from poorly differentiated primary tumors and compared it to normal esophageal epithelial cell line (HET-1A) and premalignant Barrett’s cell lines (CPA and CPB). Western blot data showed that GPC-1 was differentially expressed in cell lines and but had higher expression in PDEAC cell lines compared to HET-1A, CPA, and CPB (Figure 3A). Heparan sulfate proteoglycans like GPC-1 are known to interact with extracellular matrix cytoskeletal proteins facilitating cell division, migration, and metastasis in cancer.^28,29^ To investigate the interaction between GPC-1 and cytoskeletal proteins, we used immunofluorescence to examine the localization of endogenously expressed GPC-1 and β-tubulin in normal HET-1A cells and PDEAC cells. Normal HET-1A cells showed very weak protein expression of GPC-1. On the other hand, PDEAC ESO-26 and OE-33 cells showed strong cytoplasmic and perinuclear punctate vesicular staining patterns of GPC-1 staining (Figure 3B). Colocalization of GPC-1 and β-tubulin was observed at the tips of leading edges of the cells suggesting GPC-1 may aid in the progression of PDEAC by facilitating migratory properties of cancer cells (Figure 3B). Using proximity ligation assay (PLA), we further show that GPC-1 and Beta tubulin formed complexes in ESO-26 and OE-33 cells indicating a cross-talk between GPC-1 and cytoskeletal proteins (Figure 3C).

### Knockdown and overexpression of GPC-1 in PDEAC cell lines

To determine whether GPC-1 is a potential target in PDEAC, we utilized lentivirus system to knockdown and overexpress GPC-1. Among the PDEAC cell lines, ESO-26 and OE-33 showed the highest GPC-1 expression and SK-GT-4 lowest GPC-1 expression, therefore these cell lines were selected for functional GPC-1 knockdown (ESO-26 and OE-33) and overexpression (SK- GT4) experiments. Of the three shRNA clones, shGPC-1α showed maximum knockdown efficiency of at mRNA level reduction of GPC-1 mRNA by 96% and 92% in ESO-26 and OE-33, respectively (Figure S2A and B), whereas shGPC-1b showed maximal knockdown efficiency of GPC-1 protein reduction by 86% and 89% in ESO-26 and OE-33, respectively (Figure S2C-E). Since shGPC-1b was more effective in protein reduction, it was selected for knockdown experiments. Similarly, overexpressing GPC-1 in SK-GT4 cells resulted in > 340% and 320% increase in GPC-1 mRNA and protein levels, respectively (Figure S2F-H). No difference in GPC-1 protein or mRNA expression was seen in scrambled or empty vector control when compared to wild type PDEAC cells.

### Glypican 1 mediates growth and proliferation in esophageal cancer

**Figure 4b.**
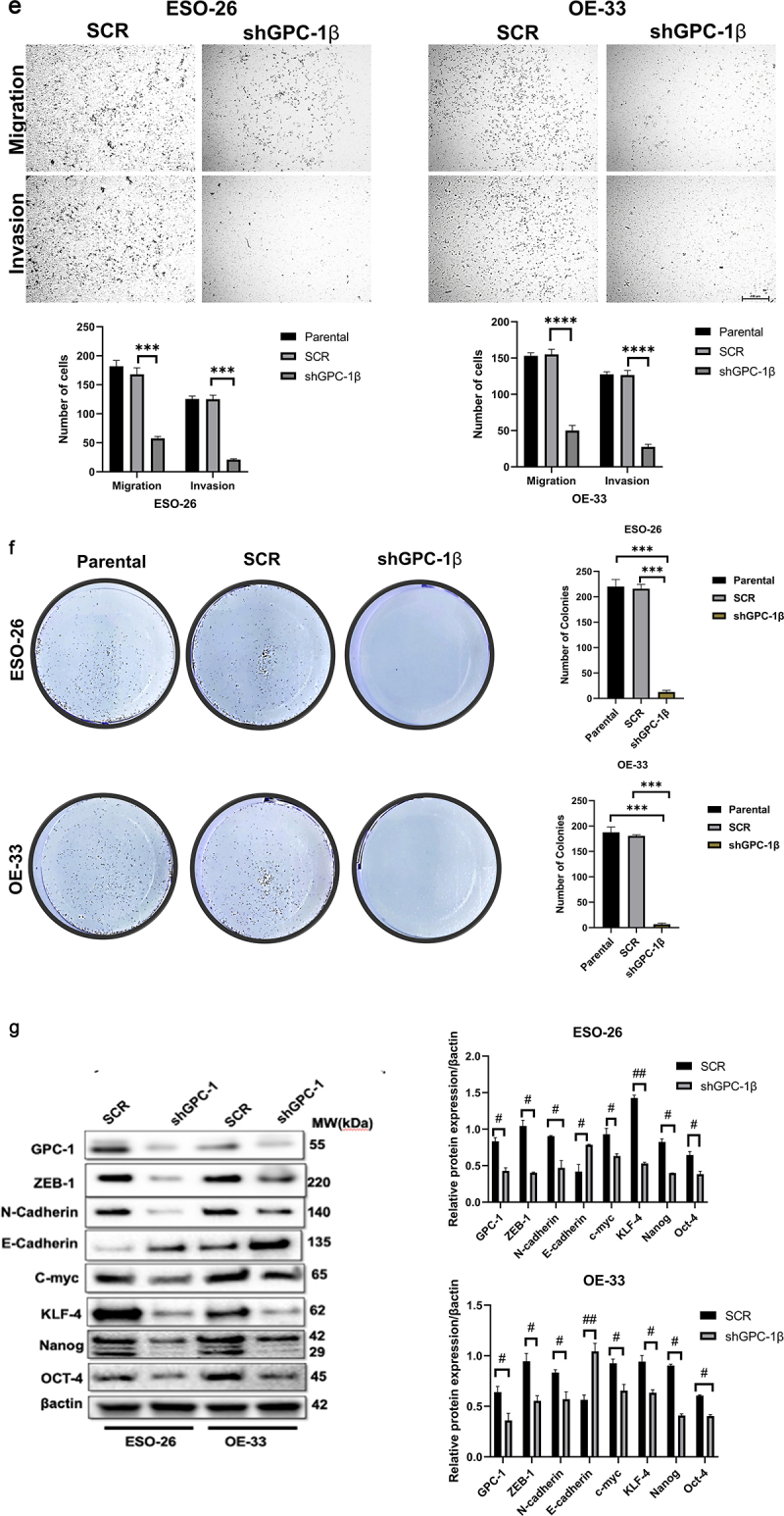
Continue

A Gene Set Enrichment Analysis technique was employed to explicate the physiological pathways that are regulated by GPC-1 in EAC cells. The current inquiry adopted the Gene Set Enrichment Analysis (GSEA) approach to evaluate the gene expression data pertaining to 150 esophageal adenocarcinoma (EAC) specimens procured from the University of California, Santa Cruz (UCSC) Xena dataset included in The Cancer Genome Atlas (TCGA). The primary objective was to ascertain the mechanistic fundamentals of the biological processes linked with GPC-1. The results of the analyses unveiled a significant association between the GPC-1 gene and fundamental molecular pathways closely tied to cell cycle, actin cytoskeleton regulation, and cancer pathways (Figure 4A). This finding accordingly alludes to a plausible implication of GPC-1 in controlling cancer-related cell growth and invasion. Having established GPC-1 knockdown cell lines, we elucidated the role of GPC-1 in the viability and proliferation of PDEAC cells. Cell viability was assessed using CCK-8 assay. As shown in Figure 4B, knockdown of GPC-1 reduced cell viability in both ESO-26 and OE-33 cells in a time-dependent manner. The wound-healing assays conducted at time points of 0 and 72 h showed that GPC-1 knockdown was significantly linked to the inhibition of primary wound closure in contrast to the scrambled control transfection (Figure 4c, d). Transwell chambers were used for migration and invasion studies. Inhibition of GPC-1 significantly decreased the number of migrating and invading cells compared to scramble control (Figure 4E). Anchorage dependent colony formation ability of ESO-26 and OE-33 was also significantly decreased after GPC-1 knockdown compared to scramble control (Figure 4F). There is substantial evidence linking epithelial mesenchymal transformation (EMT) and stemness of cancer cells to metastasis and invasion of cancers.^30,31^ Changes in molecular markers including decreased expression levels of epithelial protein E-cadherin and increased expression levels of mesenchymal protein N-cadherin and vimentin are hallmarks of EMT. Our data indicated that knockdown of GPC-1 significantly reduced expression of mesenchymal markers such as ZEB-1, N-cadherin, and c-myc while expression of epithelial marker E-cadherin was increased. Knockdown of GPC-1 also reduced the expression of key stem cell markers KLF-4, Oct-4, Nanog (Figure 4g). Collectively, these results indicate that GPC-1 plays a key role in the progression of esophageal cancer by promoting migration, cell viability, EMT, and stemness of PDEAC cells.
**Figure 4a.**
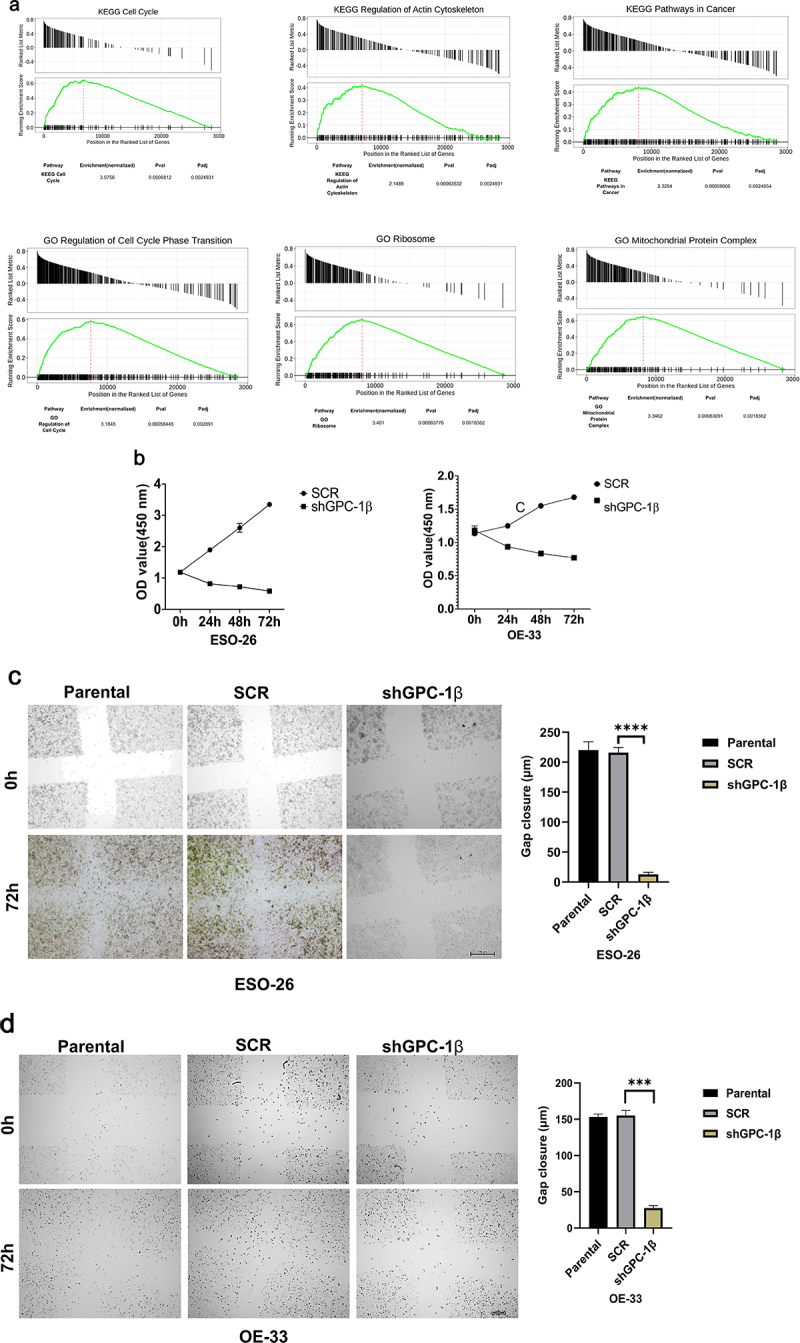
Glypican 1 mediates growth and proliferation in esophageal cancer (a) the results of the Gene Set Enrichment Analysis (GSEA) identify pathways that exhibit a significantly impactful relationship of GPC-1 in EAC. (b) Stably expressing SCR and shGPC-1β expressing cell lines ESO-26 and OE-33 cells were evaluated for cell viability by CCK-8 assay at various time points. (c-d) Light microscopy images (magnification 20×) of scratch assay taken at 0 and 72 h after transfection. (e)light microscopy images (magnification 20×) of Transwell assay in either uncoated (for migration) or Matrigel-coated (for invasion) polycarbonate 8 µm chambers. Migrated cells at the bottom of inserts were stained with 0.1% crystal violet. (f) ESO-26 and OE-33 cells were transfected with scramble shRNA and shGPC-1β and grown for 14 days, stained with crystal violet, and the number of colonies was counted. (g) Western blot analysis (left) and densitometric analysis(right) of key proteins in EMT and stemness. Bar charts represent mean ±SD, *n* = 3. ns; not significant, *p < .05,**p < .01, ***p = .002, ****p < .001.

### PDEAC cells show constitutively activated PI3K/Akt signaling driven by GPC-1

**Figure 5.**
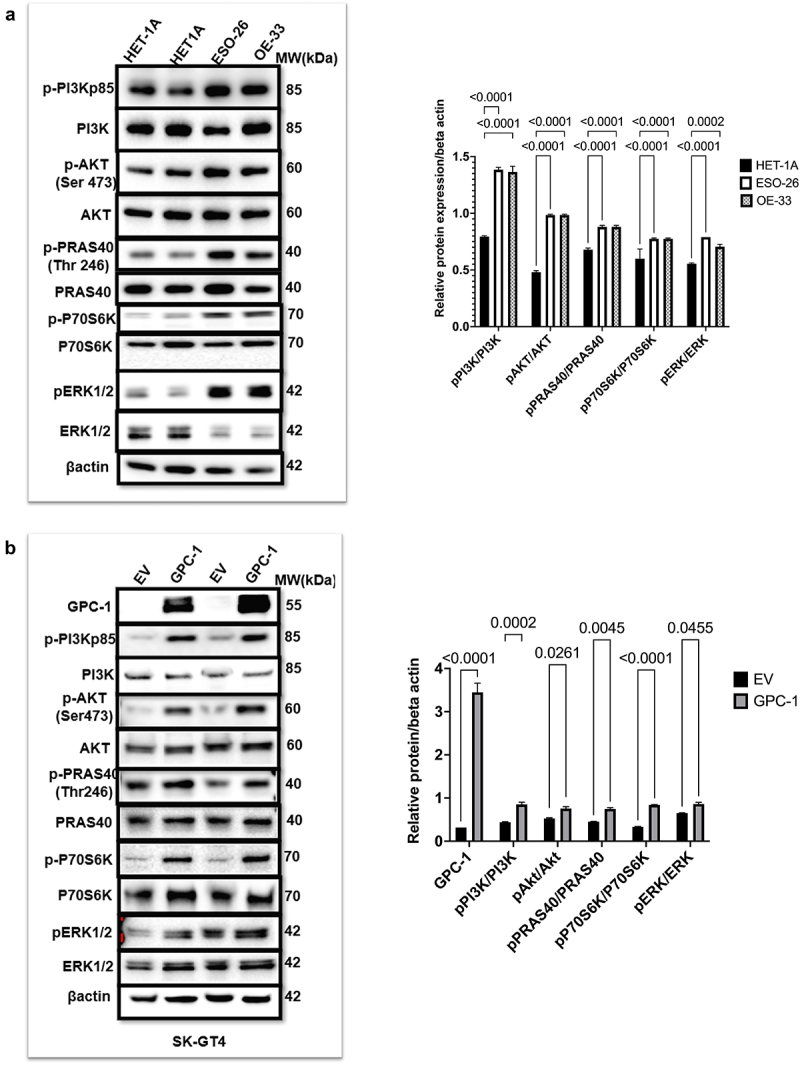
PDEAC cells have constitutively activated PI3K/Akt signaling driven by GPC-1 (a) Western blot analysis of key proteins of PI3K/Akt pathway was probed in normal HET-1A and PDEAC cell lines (ESO-26 and OE-33 cells). (b) Western blot analysis of PI3K/Akt pathway proteins after overexpression of GPC-1 in SK-GT4 cells. Bar chart of densitometry analysis of the ratio of phosphorylated to non-phosphorylated protein normalized to β-actin. Data represent mean ±SD, *n* = 3. EV, empty vector.

It is known that activation of PI3K/Akt pathway plays a crucial role in progression and metastasis of esophageal cancer.^32^ To investigate the potential contribution of the PI3K/AKT pathway in poorly differentiated esophageal adenocarcinoma, we compared the expression of PI3Kp85 (regulatory subunit), Akt and its downstream substrates PRAS40 and p70S6K in normal HET-1A cells and PDEAC cell lines ESO-26 and OE33. Western blot data showed that the two PDEAC cell lines showed a significantly higher expression of p-PI3Kp85, p-Akt (Ser473), p-PRAS40(Thr246), p-ERK1/2 and p-p70S6K compared to normal epithelial HET-1A(Figure 5a). Based on the human tissue tumor and cell-line data described earlier, we hypothesized that since GPC-1 is upstream of PI3K/Akt overexpressing GPC-1 should theoretically increase PI3K/Akt expression. To verify this hypothesis, we overexpressed GPC-1 in SK-GT4 cell line and probed for proteins of PI3K/Akt pathway. Western blot data (Figure 5b) showed that forced overexpression of GPC-1 in SK-GT4 cells resulted in significantly increased levels of p-PI3Kp85, p-Akt (Ser473), p-PRAS40(Thr246), p-ERK1/2 and p-p70S6K indicating that GPC-1 is a positive effector of PI3K/Akt signaling and may have a crucial role in tumorigenesis.

### Upregulated GPC-1 in PDEAC promotes resistance to Pictilisib

**Figure 6.**
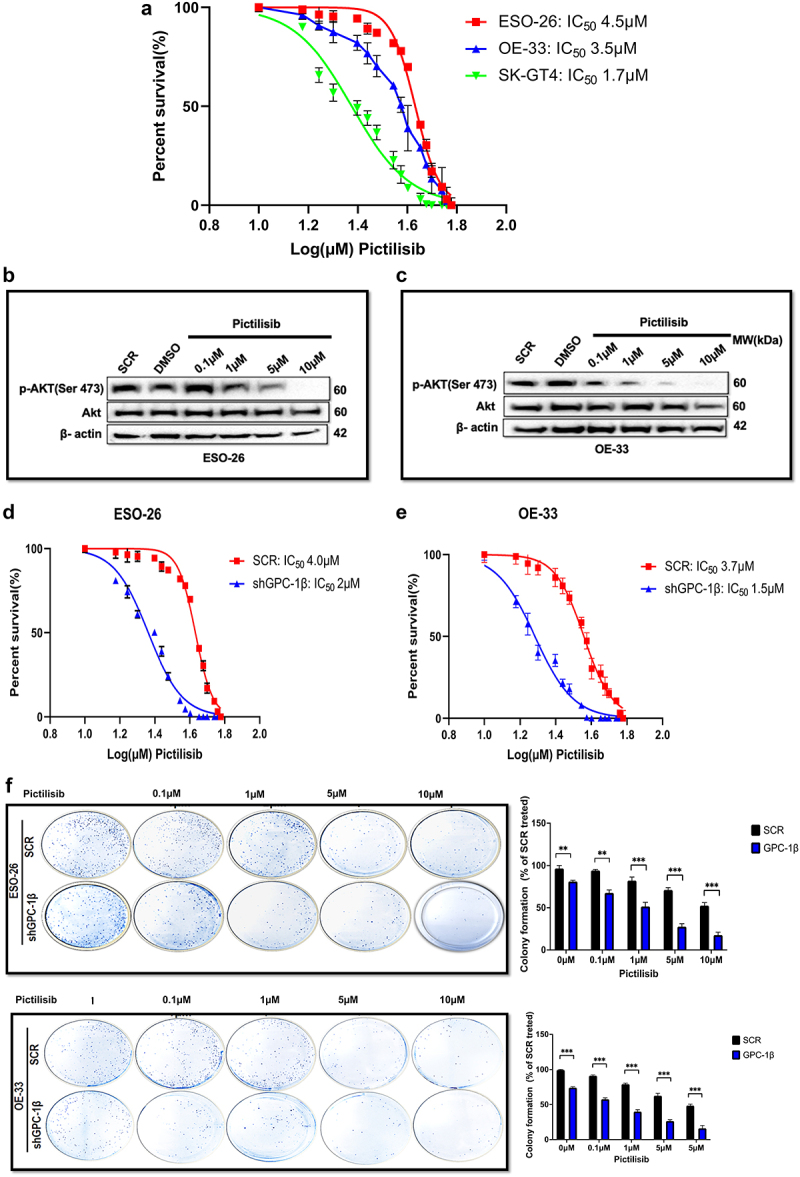
Upregulated GPC-1 expression promotes resistance to Pictilisib treatment. Cell viability was assessed using CCK-8 assay with treatment with Pictilisib (0.1 to 10 μM) for 48 h in (a) ESO-26 (b)OE-33 and (c) SK-GT4 cells. (d-e) Western blot analysis showing expression of *p*-Akt (Ser473) with varying concentrations of Pictilisib. Beta-actin was used as a loading control. (f- g) GPC-1*^High^* stably expressing SCR and shGPC-1β expressing cell lines were treated with a range of concentrations (0.1 to 10 μM) of Pictilisib. CCK-8 assay was used to measure cell viability and IC_50_ 48 h after treatment. The IC_50_value of ESO-26shSCR and ESO-26shGPC-1β 3.54 µM and 0.32 µM respectively. The IC_50_value of OE-336shSCR and OE-33shGPC-1β 1.34 µM and 0.309 µM respectively. (h) Clonogenic survival assays in GPC-1*^High^* stably expressing SCR and shGPC-1β expressing cell lines (ESO-26 and OE-33) treated with the indicated doses (µM) of Pictilisib for 48 hours. Media was changed and cells were then cultured for 14 days without inhibitor, fixed with 4% paraformaldehyde and stained with 0.1%crystal violet. Colonies were counted and expressed as a percentage of the SCR control. **p < .01; ***p < .001.

Having shown that PI3K/Akt pathway is constitutively upregulated in PDEAC, inhibiting this signaling cascade has attractive potential in PDAC treatment. For this study, we chose Pictilisib, a pan PI3K inhibitor, which targets the most upstream of PI3K/Akt pathway and has shown promising results in clinical trials.^33,34^ However, the development of drug resistance and toxicity has limited its widespread use.^35,36^ We hypothesize that higher expression of GPC-1 in PDEAC could be one of the key contributors to Pictilisib resistance. To test this hypothesis, we treated GPC-1^*High*^ (ESO-26 and OE-33) and GPC-1^Low^ (SK-GT4) expressing cells with a range of Pictilisib concentration (0.1 μM to 10 μM) for 48 h and measured cell viability with CCK-8 assay. Susceptibility to Pictilisib measured by its IC_50_ correlated with the level of GPC-1 expression. GPC-1^high^ cell lines showed greater resistance to Pictilisib (IC_50_:4.5 µM for ESO-26 and 3.5 µM for OE-33 cells) compared to GPC-1^low^ (IC_50_:1.7 µM for SK-GT4) expressing cells (Figure 6a). These findings are consistent with the increased sensitivity of SK-GT4 cells to PI3K inhibitor by virtue of activating mutation of PIK3CA in this cell line. Western blot analysis was performed to further examine the effect of Pictilisib on phosphorylation of Akt (Ser473) in GPC-1^High^ expressing ESO-26 and OE-33 cells. Western blotting results indicated that Pictilisib inhibited p-Akt (Ser473) expression in a dose-dependent manner in ESO-26(Figure 6b and OE-33 cells (Figure 6c) with complete abrogation of p-Akt (Ser473) signal at a dose of 10 µM in both cell lines. One of the key advantages of combinatorial therapy is the ability to inhibit multiple cell proliferation and survival pathways. Based on current literature and data presented above, we show that GPC-1 is a positive effector on PI3K/Akt pathway. Consequently, it is a plausible inference that concurrent implementation of genetic suppression of GPC-1 and the utilization of a chemical inhibitor targeting the PI3/Akt pathway (Pictilisib) could serve as a promising strategy for mitigating resistance and promoting the efficacy of therapeutic interventions. To examine the efficacy of this combinatorial therapy, we treated GPC-1^High^ stably expressing control shGPC-1 and shGPC-1β expressing cell lines with a range of concentration of Pictilisib. CCK-8 assay was used to measure cell viability and IC_50_ 48 h after treatment. Combinatorial treatment reduced IC_50_ in ESO-26 and OE-33 cells from 4 µM and 3.7 µM to 2 µM and 1.5 µM, respectively (Figure 6d, e). The extended culture of cells treated with shGPC-1 and Pictilisib exhibited a marked reduction in colony outgrowth due to sensitization toward PI3K inhibition (Figure 6F). These results suggest that upregulated GPC-1 expression confers resistance to Pictilisib treatment and silencing GPC-1 as a combinatorial treatment overcomes resistance to Pictilisib in PDEAC cells.

### GPC-1 knockdown sensitized GPC-1^high^ expressing PDEAC cells to antitumor effects of low dose Pictilisib via downregulating Akt signaling

**Figure 7.**
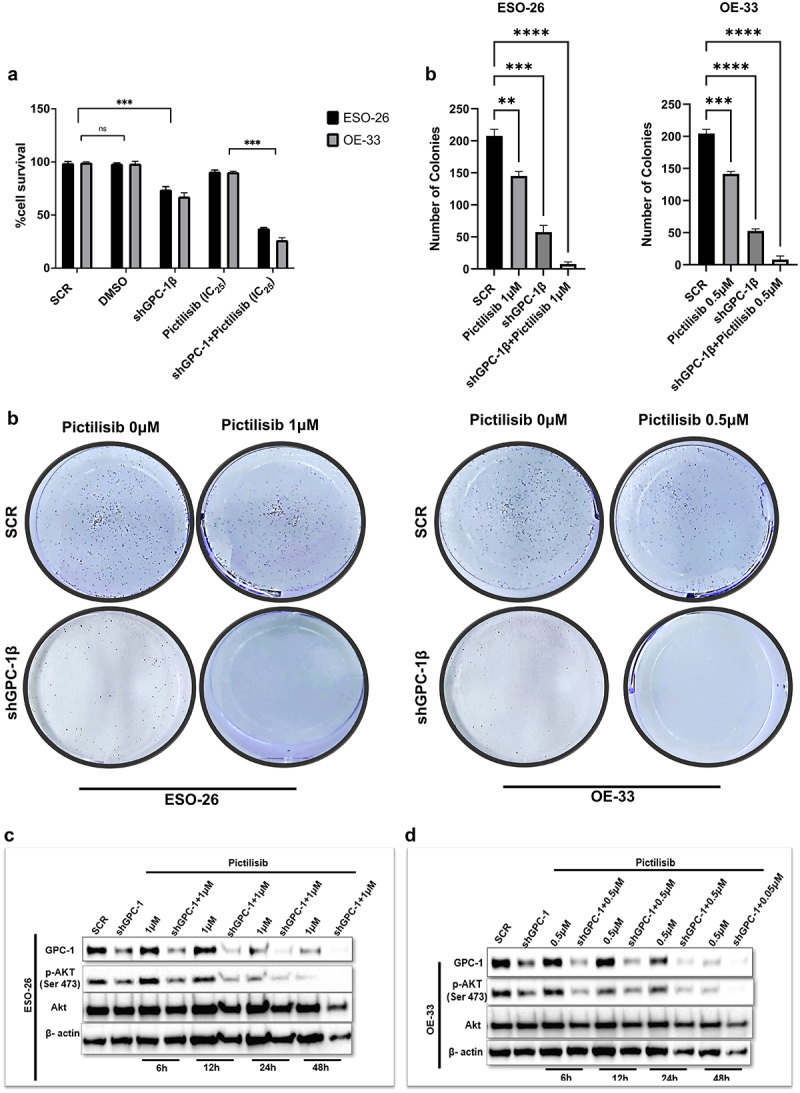
GPC-1knockdown sensitized GPC-1*^high^* expressing PDEAC cells to antitumor effects of low dose Pictilisib via downregulating Akt signaling. (a) GPC-1*^High^* stably expressing SCR and shGPC-1β expressing cell lines were treated with low dose Pictilisib (IC_25_) for 48 h and CCK-8 assay was used to measure cell viability. (b) Clonogenic survival assays in GPC-1*^High^* stably expressing SCR and shGPC-1β expressing cell lines (ESO-26 and OE-33) treated with Pictilisib (IC25, µM) for 48 hours. Media was changed, cells were cultured for 14 days without inhibitor, fixed with 4% paraformaldehyde and stained with 0.1%crystal violet. Colonies were counted and expressed as a percentage of the SCR control. (c-d) Western blot showing expression of *p*-AKT(Ser473) levels at shown time points after treatment of GPC-1*^High^* shGPC-1β expressing cell lines (ESO-26 and OE-33 cells) with low dose Pictilisib (IC_25_). Combinatorial treatment with low dose Pictilisib synergistically reduced p-AKT level in both cell lines at 48 h. ns, not significant; ***p < .001.

Exploring combinatorial therapy in cancer treatment allows dose reduction of drugs without compromising the dose-dependent inhibition of proliferation and cell survival pathways. Mechanistically, Pictilisib reduces phosphorylation of Akt at Serine 473 residue which is considered as a reliable readout of Akt activity. Here we next analyzed the effect of co-administration of low-dose Pictilisib (IC_25_) and shGPC-1β on viability, colony formation and phosphorylation of Akt (Ser473) in GPC-1^High^ expressing cells. CCK-8 assay showed that exposure of ESO-26 and OE-33 cells to IC_25_ of Pictilisib combined with shGPC-1β for 48 h resulted in a significant reduction of viable cells by 23% and 21%, respectively, denoting a statistically significant effect compared to controls (Figure 7a). Low dose Pictilisib in combination with shGPC-1β markedly reduced colony outgrowth, with OE-33 cells showing more sensitivity to the combination compared to ESO-26 cells (Figure 7b). Western blot analysis showed a time dependent significant reduction of p-Akt (Ser473) expression with the combinatorial treatment explaining the observation of amplified effect of combinatorial treatment on cell viability (Figure 7c, d).

### Silencing GPC-1 alone or in combination with Pictilisib effectively downregulates PI3K/Akt/ERK pathway

**Figure 8.**
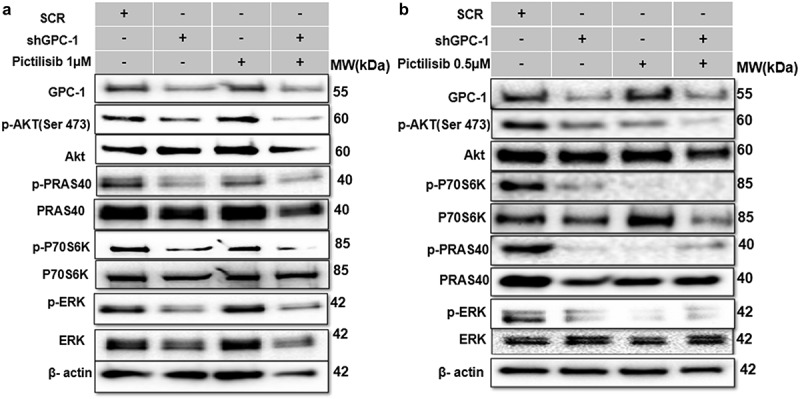
Silencing GPC-1 alone or in combination with Pictilisib effectively downregulates PI3K/Akt/ERK pathway. (a-b) GPC-1*^High^* stably expressing SCR and shGPC-1β expressing cell lines were treated with low dose Pictilisib (IC_25_) for 48 h and cell lysates were subjected to western blot analysis with antibodies for GPC-1, *p*-AKT(Ser473), total Akt, *p*-PRAS(Thr246), total PRAS, p-P70S6K, total P70S6K, p-ERK1/2 and total ERK1/2. Beta-actin was used as a loading control.

Whether GPC-1 knockdown alone or its combination with low-dose Pictilisib could effectively suppress PI3K/Akt/ERK pathway was investigated with western blot analysis of key protein markers of the pathway. Knockdown of GPC-1 or Pictilisib (IC_25_) alone significantly decreased phosphorylation of Akt (Ser473), PRAS(Thr246), P70S6K, and ERK1/2 in both ESO-26 and OE-33 cells (Figure 8a, b). Combining GPC-1 knockdown with Pictilisib further reduced phosphorylation of downstream proteins of PI3K/Akt/ERK in both cell lines.

### Knockdown of GPC-1 enhanced Pictilisib induced apoptosis and cell cycle arrest in G0/G1 phase in PDEAC cells

**Figure 9b.**
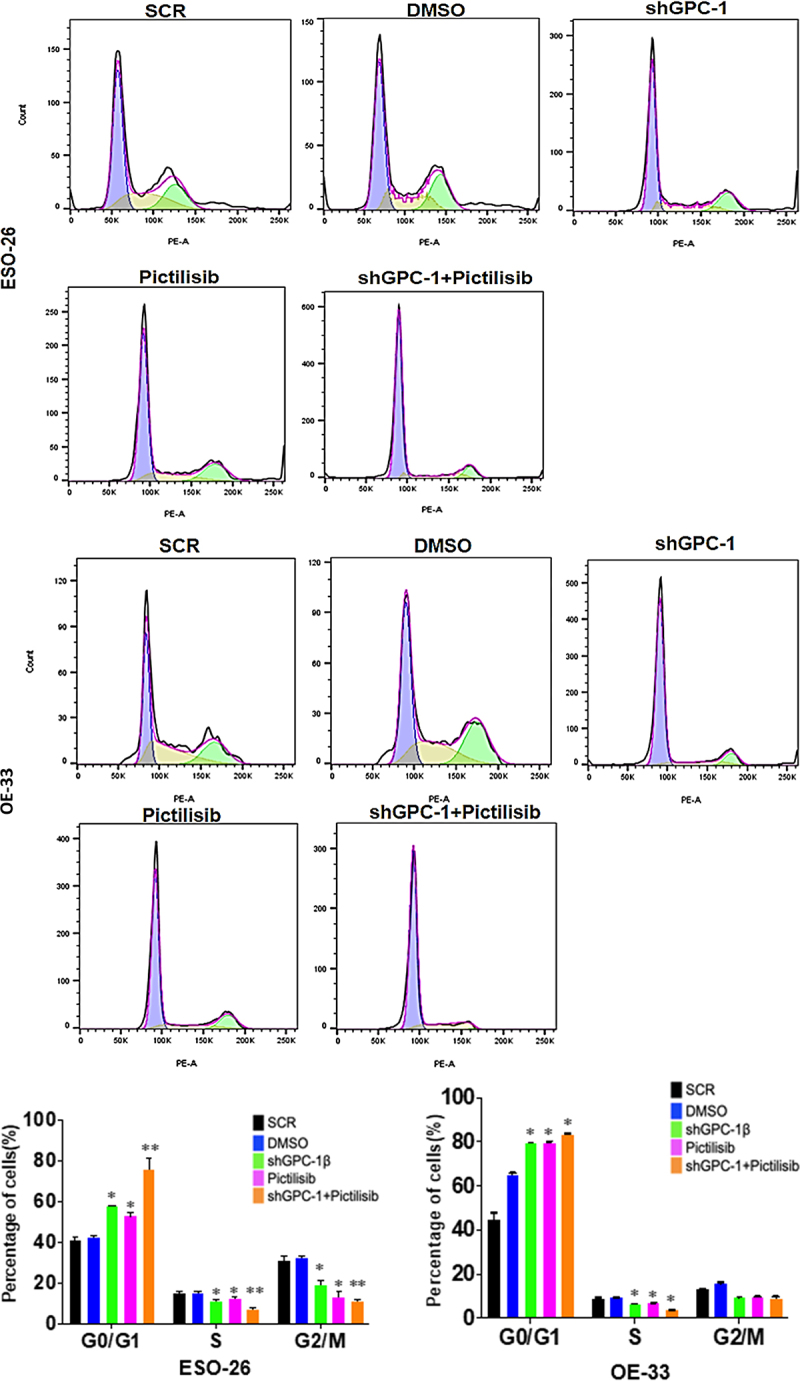
Continue

To further elucidate the mechanism of growth suppression by combinatorial treatment of GPC-1 knockdown and Pictilisib, we investigated apoptosis using Annexin-V/FITC staining by flow cytometry. As shown in Figure 9a, no difference in apoptosis was seen among scrambled control shRNA and vehicle (DMSO) in ESO-26 and OE-33 cells. In contrast, GPC-1 knockdown and Pictilisib both induced significant apoptosis. The relative percentage of apoptotic cells markedly increased after combination treatment of shGPC-1β and Pictilisib. These findings support that knockdown of GPC-1 when combined with Pictilisib induces greater apoptosis of PDEAC cells. Cell cycle distribution analysis was performed to investigate the effects of combination therapy on proliferation other than apoptosis. After 48 h hours of treatment with shGPC-1β, IC_25_ Pictilisib or both, cell cycle was analyzed by propidium iodide (PI) flow cytometry. There were no changes in scramble control or vehicle groups with the treatment. Compared to scramble control both shGPC-1β and Pictilisib group showed a greater percentage of cells in G0/G1 phase and a shortened S phase (Figure 9b). Combining shGPC-1β and Pictilisib resulted in a greater percentage of cells arrested in G0/G1 phase and significantly short S phase demonstrating the knockdown of GPC-1β with Pictilisib treatment results in an amplified arrest of cells in G/G1 phase.
**Figure 9a.**
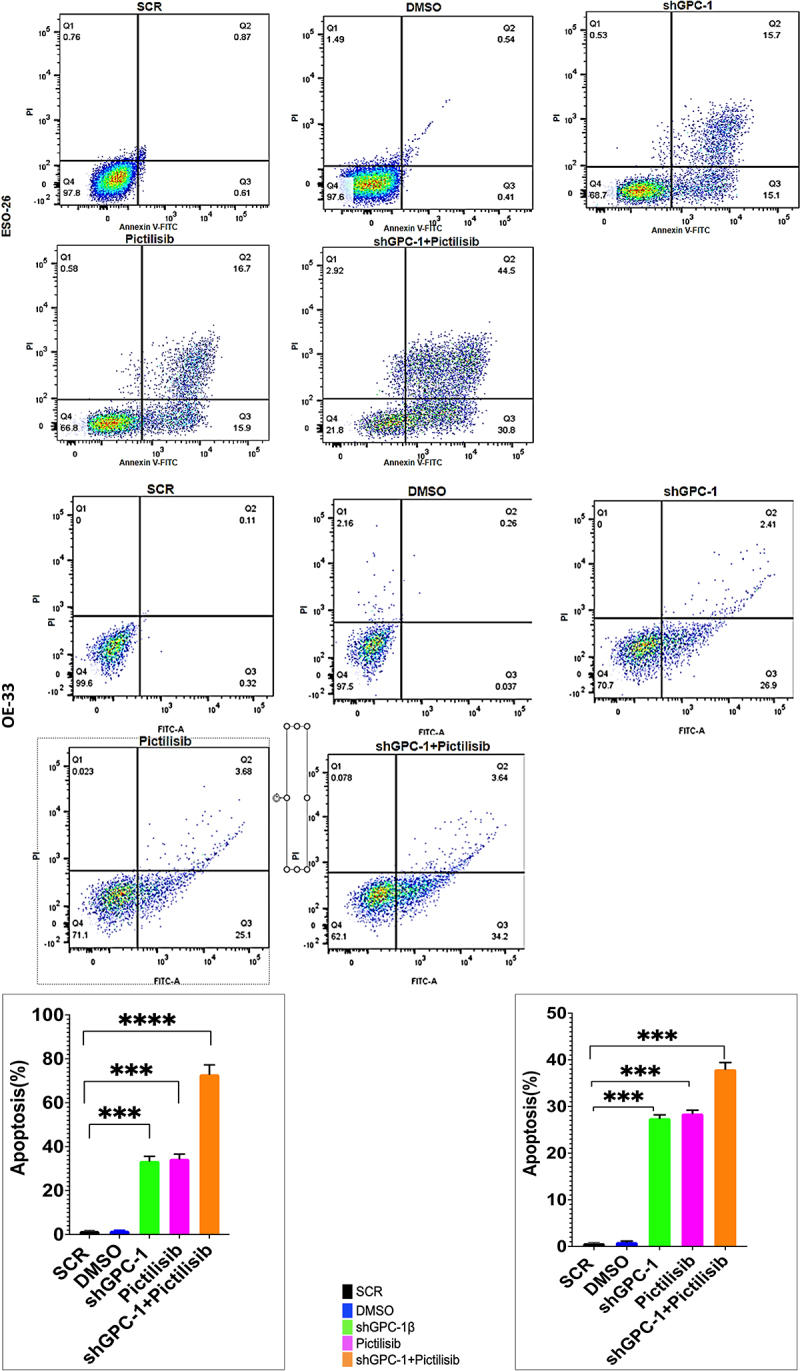
Knockdown of GPC-1 enhanced Pictilisib induced apoptosis and cell cycle arrest in G0/G1 phase in PDEAC cells. (a) GPC-1*^High^* stably expressing SCR and shGPC-1β expressing cell lines were treated with low dose Pictilisib (IC_25_) for 48 h and evaluated for apoptosis using Annexin V/FITC using flow cytometry. the downregulation of GPC-1 synergized with low-dose Pictilisib to induce apoptosis in ESO-26 and OE-33 cells. (b\) GPC-1*^High^* stably expressing SCR and shGPC-1β expressing cell lines were treated with low dose Pictilisib (IC_25_) for 48 h and evaluated for the cell cycle stage using Propidium iodide staining followed by flow cytometry Data represents *n* = 3, mean ± SD; ordinary one ANOVA with multiple comparisons, *, P < 0.05, SCR, negative scrambled control; shGPC-1β, GPC-1 knockdown plasmid; FITC, fluorescein isothiocyanate.

### Combination of GPC-1 knockdown and Pictilisib results in greater tumor growth inhibition in-vivo

**Figure 10b.**
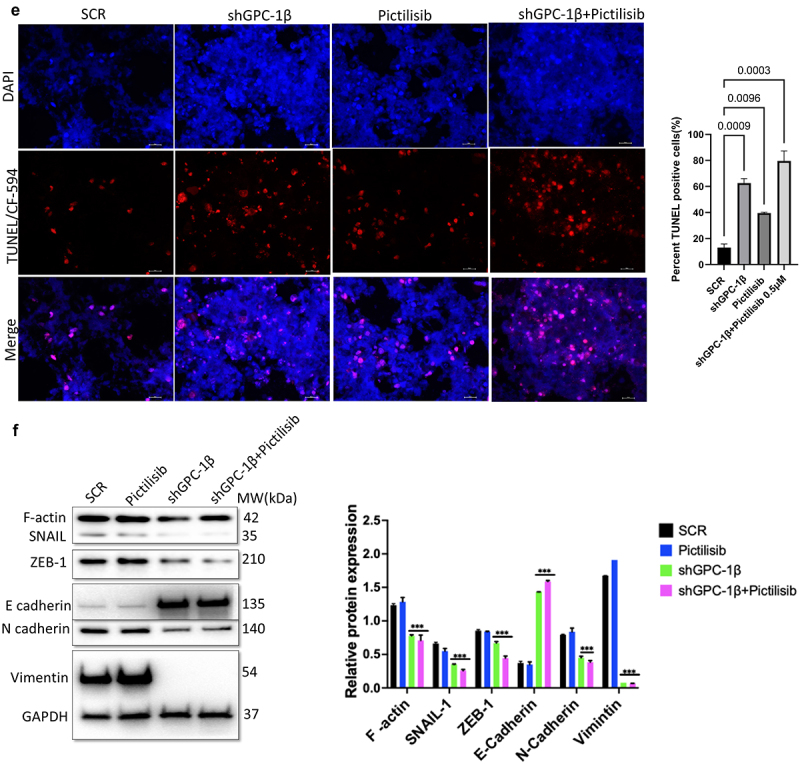
Continue

Notwithstanding the robust suppressive impact on cell proliferation attributed to shGPC-1β-mediated knockdown and its combination with Pictilisib, nonetheless the pertinent clinical query persists as to whether these observed synergistic outcomes in vitro can be transposed to efficacious in vivo antineoplastic effects. We selected ESO-26 cells for in vivo tumor experiments in BALb/c nude mice since this cell line expressed higher GPC-1 levels and showed greater resistance to Pictilisib compared to OE-33 cells. shGPC-1 alone showed excellent reduction in tumor burden when compared to Pictilisib, moreover tumor growth was abrogated significantly when shGPC-1 was combined with Pictilisib (Figure 10a). Synergistic effect with combination therapy was seen in reducing tumor growth reflected by significantly reduced mean tumor volumes (Figure 10b) and weight (Figure 10c). Immunohistochemistry performed for Ki-67 and TUNEL showed markedly lower Ki-67 staining and increased percentage of TUNEL positive cells in xenografts of mice that were treated with shGPC-1β as well those treated with its combination with Pictilisib (Figure 10d, e). Western blot analysis for EMT proteins revealed a significantly reduced expression of mesenchymal markers and an increase in epithelial markers with shGPC-1β alone or its combination with Pictilisib (Figure 10f). These findings align with the observed cellular mortality and clonogenic evidence, providing additional support to the notion that the combination of silencing GPC-1 with PI3K inhibition results in a synergistic impact in inhibiting EAC growth.
**Figure 10a.**
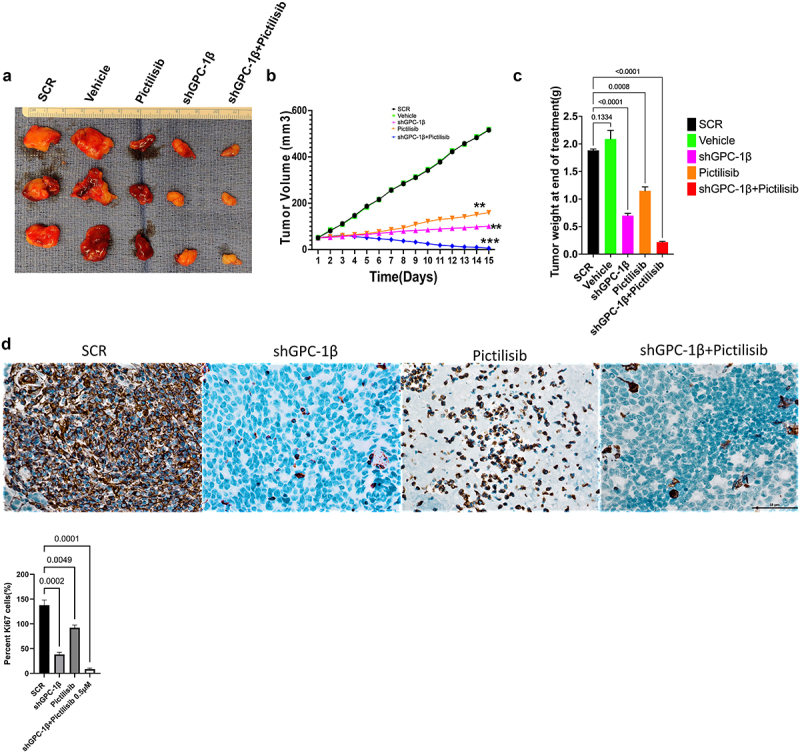
Combination of GPC-1 knockdown and Pictilisib results in greater tumor growth inhibition in-vivo. (a) Knockdown of GPC-1 notably inhibited the growth of ESO-26 which was more significant after combination with Pictilisib. (b-c) Tumor volumes and weight of xenografts were significantly reduced with combination treatment. (d) Immunohistochemistry staining for Ki67. Scale bar: 10 µm. (e) Fluorescent images of xenograft tissue sections stained for TUNEL staining using a TUNEL/CF-594 staining kit. Blue denotes DAPI, red denotes CF-594 and pink denotes merged condensed chromatin within the nucleus. Scale bar: 50 µm. (f) Western blot analysis of EMT proteins at the treatment endpoint of 15 days. Data represents the mean ± SD of 4 animals. **p < .01; ***p < .001.

## Discussion

Esophageal cancer is the sixth leading cause of cancer related mortality worldwide.^1^ Most EAC patients in the United States are diagnosed at a late stage (Stage III or IV) and have a poor response to therapy, resulting in a five-year survival rate of only 23%. Despite recent improvement in treatment regimens, patients develop chemoresistance resulting in poor clinical outcome. New therapeutic targets and biomarkers for early diagnosis and targeted treatment are urgently needed to improve clinical outcomes in EAC patients. Accumulating evidence suggests that activation of the PI3K/Akt pathway is essential in the initiation and progression of several cancers including esophageal cancer. Next-generation sequencing has shown that mutations in this pathway are present at a higher rate in locally advanced poorly differentiated tumors. PI3K/Akt pathway controls a vast array of biological and physiological processes including but not limited to cell growth, epithelial mesenchymal transition (EMT), angiogenesis, metastasis and multidrug resistance. Thus, genetic or chemical inhibition of this pathway could be a logical source for further investigations into combinatorial efficacy with other chemotherapeutic agents. A significant development over the past few years has been the progression of small molecule PI3K inhibitors into clinical trials. Pictilisib also known as GDC-0941 is a highly selective orally active class I PI3K inhibitor that has shown promising efficacy with tolerable side effects in preclinical advanced breast cancer and solid tumors, however in human trials clinical regression of tumors was not seen. Possible explanations include feedback activation of Akt (Ser473) phosphorylation by mTOR or RAF/RAS/MEK signaling which are unaffected by PI3K inhibition alone.^30–32^ Moreover > 90% inhibition of p-Akt is required to effectively see the benefits of PI3K inhibitors, which would require very high dose in toxic range.^33^ To overcome these limitations, we embarked on finding augmenters of response to Pictilisib in PDEAC. Glypican 1 is a member of the heparan sulfate proteoglycan family which is expressed on cell surface and extracellular matrix in various tissues.^34^ Recently we and others have shown that increased expression of GPC-1 correlated with advanced TNM stage, lymphovascular spread and poor differentiation in EAC.^18^ There is also new evidence that GPC-1 plays an important role in cell proliferation and contributes to progression and chemoresistance in many cancers via regulating PI3K/Akt/mTOR signaling.^22,35^ In the present study we selected a cohort of poorly differentiated esophageal adenocarcinoma (PDEAC) and found overexpression of GPC-1 in 71% of tumor specimens compared to normal tissue. The present study investigated the potential correlation between augmented expression of GPC-1 and the histopathological response toward neoadjuvant chemotherapy. The clinical findings indicated a notable correlation between intensified GPC-1 expression and impairments in response to chemotherapeutic treatments. Henceforth, we endeavored to elucidate the mechanism of the association in vitro. The experimental findings indicate that suppressing the expression of GPC-1 in PDEAC cells led to a significant reduction in their ability to survive, proliferate, migrate, and invade, suggesting a potential oncogenic role for GPC-1. Pictilisib inhibited up to varying degrees the proliferation of multiple PDEAC cell lines and resistance to Pictilisib correlated with degree of GPC-1 expression. GPC-1^high^ (ESO-26 and OE-33) cells were more resistant to Pictilisib compared to GPC-1^low^ expressing SK-GT4 cells. Cells that were resistant to Pictilisib became sensitized when GPC-1 was silenced and showed significantly reduced IC_50_ and clonogenic survival. This finding has significant implications in the study of chemoresistance and suggests a potential role of GPC-1 in promoting resistance to PI3K inhibitor treatment. Taken as a whole, these findings suggest that the upregulation of GPC-1 expression may be specifically implicated in conferring resistance to PI3K inhibitors or conventional chemotherapy. To further clarify the mechanisms of GPC-1 effect on PDEAC cells, we analyzed phosphorylation of key proteins of PI3K/Akt/ERK signaling after GPC-1 knockdown, Pictilisib or combination of the two. We show GPC-1 knockdown alone or combined with Pictilisib reduced proliferation, and colony outgrowth. More importantly, we showed that combinatorial therapy of GPC-1 knockdown with low-dose Pictilisib (IC_25_) augmented antitumor efficacy to achieve cell death and tumor regression. Our data convincingly shows a strong inhibitory effect of GPC-1 knockdown on PI3K/Akt/ERK axis by reducing the phosphorylation of p-Akt (Ser473), p-PRAS40, p-P70S6, and p-ERK in GPC-1^High^ expressing ESO-28 and OE-33 cells. Our data also show a direct effect of GPC-1 knockdown on cell cycle progression. During the cell cycle, several factors must be carefully controlled. Losing control of these factors may ultimately result in tumor development. Flow cytometry data using PI staining demonstrated that knockdown of GPC-1 and Pictilisib inhibited growth predominantly by causing cell cycle arrest in G0/G1 phase in GPC-1^High^ expressing PDEAC cells, which is consistent with other reports.^36^ This inhibitory effect was even more pronounced when shGPC-1β and Pictilisib were used in combination. Similarly, analysis of apoptosis with Annexin V/FITC and western blot indicated GPC-1 knockdown sensitized ESO-26 and OE-33 cells to Pictilisib induced apoptosis which is in agreement with previous reports which have shown that apoptosis response in PI3K/Akt inhibition is mediated by increased mitochondrial membrane damage and caspase 3 activation.^37,38^ Collectively, the results indicate that by introducing GPC-1 blockade to chemotherapy regimens, the number of cells that are immune to the drug could be reduced, thereby improving the outcomes of therapy. The findings of the in vitro study were corroborated by the in vivo investigation conducted on murine xenograft model. The results showed that the use of shRNA to suppress GPC-1 had a significant impact on restraining tumor growth, a result that was further augmented in the presence of Pictilisib. The association between the substantial reduction of Ki-67 expression and the notable increase in TdT-mediated dUTP-biotin nick end labeling (TUNEL)-positive cells is the principal cause of the synergetic effect observed in inhibiting the growth of PDEAC cells. Epithelial mesenchymal transformation is a complex multistep process by which epithelial cells transition to invasive mesenchymal cells by losing their apical-basal polarity and cell-cell adhesion. Cells that go through EMT have lower expression of genes that are related to epithelial cells, such as E-cadherin, and ZO-1 and higher levels of genes related to mesenchymal cells, such as N-cadherin, vimentin, and fibronectin.^39^ Our in vivo experiments showed that knockdown GPC-1 alone or when combined with Pictilisib significantly inhibited EMT by upregulating E-cadherin and down regulating F-actin, N-cadherin, ZEB1, vimentin, and SLUG. The present study concludes that the outcomes of both in vitro and in vivo experiments support the notion that GPC-1 knockdown attenuates the resistance of EAC cells to PI3K inhibitor by impeding EMT, stemness, inducing G0/G1cell cycle arrest, and facilitating apoptotic cell death. To the best of our knowledge, this study represents the inaugural investigation proposing that GPC-1 demonstrates potential as a therapeutic target in augmenting the chemosensitivity of therapy resistant poorly differentiated esophageal adenocarcinoma.

## Supplementary Material

Supplemental Material

Supplemental Material

Supplemental Material

Supplemental Material

Supplemental Material

## Data Availability

The data that support the findings of this study are available from the corresponding author, [AP], upon reasonable request to akshay.chauhan@cuanschutz.edu.
