# Supplementary material for: Silencing Glypican-1 enhances the antitumor effects of Pictilisib via downregulating PI3K/Akt/ERK signaling in chemo-resistant esophageal adenocarcinoma
Source: Mol Cell Oncol. 2023 Aug 15;10(1):2238873. doi: 10.1080/23723556.2023.2238873 (PMC10464651; doi:10.1080/23723556.2023.2238873)
Supplement: Supplemental Material [file KMCO_A_2238873_SM3623.docx]

**Supplemental Figure S1**. Duolink proximity ligation assay for protein-protein interactions between GPC-1 and β-tubulin in ESO-26 and OE-33 cell lines. The cells were fixed and treated with Duolink assay as described (cells without treatment as negative controls). Each red spot represents a single interaction and DNA was stained with DAPI. Scale bar: 100µm.

**Supplemental Figure S2.** Efficient knockdown and overexpression of GPC-1 in PDEAC cells. (A-B) Bar chart showing real-time qPCR analysis of GPC-1 mRNA levels in ESO-26 and OE-33 cell lines after transfection with three GPC-1 shRNA constructs. (C-E) Western blot validation of GPC-1 knockdown in ESO-26 and OE-33 cell lines. Bar chart showing densitometric analysis of GPC-1 protein expression after the knockdown. (F) Bar chart of real-time qPCR analysis of GPC-1 overexpression in SK-GT4 cells with GPC-1 lentiviral transfection. (G-H) Western blot analysis of GPC-1 protein expression with overexpression plasmids. Bar charts represent mean ±SD, n=3. ns; not significant, ***p<0.001, **** p<0.0001. shGPC-1α, shGPC-1β, and shGPC-1D, GPC-1 knockdown plasmids; SCR, scramble negative control; EV, empty vector.
